# Occurrence and role of Tph cells in various renal diseases

**DOI:** 10.1186/s10020-024-00919-3

**Published:** 2024-10-11

**Authors:** Junyi Ren, Kuai Ma, Xiangheng Lu, Haoyu Peng, Jia Wang, Moussa Ide Nasser, Chi Liu

**Affiliations:** 1 School of Medicine, University of Electronic Science and Technology of China, Chengdu, China; 2 Department of Nephrology, Osaka University Graduate School of Medicine, Osaka, Japan; 3 Department of Ophthalmology, West China Hospital, Sichuan University, Chengdu, Sichuan China; 4 General Practice Center, Sichuan Provincial People’s Hospital, Sichuan Academy of Sciences, University of Electronic Science and Technology, Chengdu, 610072 China; 5 Department of Cardiac Surgery, Guangdong Cardiovascular Institute, Guangdong Provincial People’s Hospital (Guangdong Academy of Medical Sciences, Southern Medical University, Guangzhou, 510100 Guangdong China; 6 Department of Nephrology and Institute of Nephrology, Sichuan Provincial People’s Hospital, Sichuan Clinical Research Centre for Kidney Diseases, Chengdu, China

**Keywords:** Peripheral helper T cells, T follicular helper T cells, Autoimmune nephropathy, Renal diseases

## Abstract

A new population of peripheral helper T (Tph) cells has been identified and contributed to various autoimmune diseases. Tph cells can secrete interleukin-21 (IL-21), interferon (IFN) and C-X-C motif chemokine ligand 13 (CXCL13) to moderate renal disease. Moreover, Tph cells can congregate in huge numbers and immerse within inflamed tissue. Compared to Tfh cells, Tph cells express high programmed cell death protein 1 (PD-1), major histocompatibility complex II (MHC-II), C-C chemokine receptor 2 (CCR2) and C-C chemokine receptor 5 (CCR5) but often lack expression of the chemokine receptor C-X-C chemokine receptor 5 (CXCR5). They display features distinct from other T cells, which are uniquely poised to promote responses and antibody production of B cells within pathologically inflamed non-lymphoid tissues and a key feature of Tph cells. In this review, we summarize recent findings on the role of Tph cells in chronic kidney disease, acute kidney injury, kidney transplantation and various renal diseases.

## Introduction

A recently identified subset of T helper cells, referred to as T peripheral helper (Tph) cells, demonstrates heightened programmed cell death protein 1 (PD-1) expression and diminished expression of C-X-C motif chemokine receptor 5 (CXCR5) (Rao et al. 2017). This distinctive population primarily localizes within peripheral inflammatory tissues and exerts its functional effects through the modulation of various cytokines, notably interleukin-21 (IL-21) (Yoshitomi and Ueno 2021) and CXCL13 (Kobayashi et al. 2016).

Many hypotheses have been raised regarding the generation of Tph cells. To be specific, naive T cells can differentiate into Tph cells upon stimulation by transforming growth factor beta (TGF-β) (Kobayashi et al. 2016) or autoantigens presented by plasmacytoid dendritic cells (pDCs) (Caielli et al. 2019). Tph cells exhibit comparable levels of the transcription factor RAR-related orphan receptor gamma t (RORγt), characteristic of the Th17 lineage, probably suggesting a potential lineage relationship between Tph and Th17 cells (Fischer et al. 2022). Interferon-alpha (IFN-α) can suppress BCL-6 expression in Tfh cells, diminishing the expression of CXCR5 and facilitating their transition toward Tph cells (Jiang et al. 2022; Liu et al. 2021). In summary, naïve T cells, Tfh cells and other T helper cells might be able to differentiate into Tph cells.

Recent investigations underscore the crucial role of Tph cells in the pathogenesis of autoimmune kidney diseases (Huang et al. 2023). In order to alleviate autoimmune kidney diseases, certain scholarly investigations have demonstrated that an appreciably increased proportion of Tph cells is observed in the peripheral blood of patients suffering from autoimmune disorders, concomitant with elevated TIGIT expression. Likewise, the application of anti-human TIGIT agonistic monoclonal antibodies effectively curtails the activation and proliferation of Tph cells, concurrently dampening their paracrine influence on B cells. This discovery presents a promising therapeutic avenue with the potential to yield positive outcomes in managing autoimmune conditions (Kojima et al. 2023). Consequently, investigating the role of Tph cells in various autoimmune kidney diseases and identifying potential therapeutic targets within these conditions is essential.

Meticulous exploration into the multifaceted roles played by diverse Tph cell subsets in the context of autoimmune kidney ailments and the renal lesions associated with other autoimmune disorders holds profound scientific significance. This review may enhance our comprehension of the etiology underlying autoimmune diseases and, in turn, substantiate the development of novel therapeutic agents, offering a robust framework for progress in this domain.

## General molecular features

Tph cells exhibit notable molecular characteristics characterized by elevated PD-1 expression and reduced CXCR5 expression (Huang et al. 2023). In various autoimmune kidney diseases, distinct phenotypes may emerge, including ICOS^+^ cells (Edner et al. 2020), Foxp3- cells (Pontarini et al. 2020a, b), and CD45RA- cells (Makiyama et al. 2019a, b). Additionally, Tph cells exhibit heightened expression of inflammatory chemokine receptors, including C-C chemokine receptor 2 (CCR2), C-X3-C motif chemokine receptor 1 (CX3CR1), and C-C chemokine receptor 5 (CCR5) (Zhang et al. 2019; O’Connor et al. 2015; Balistreri et al. 2007; Vietinghoff and Kurts 2021).

**Fig. 1:**
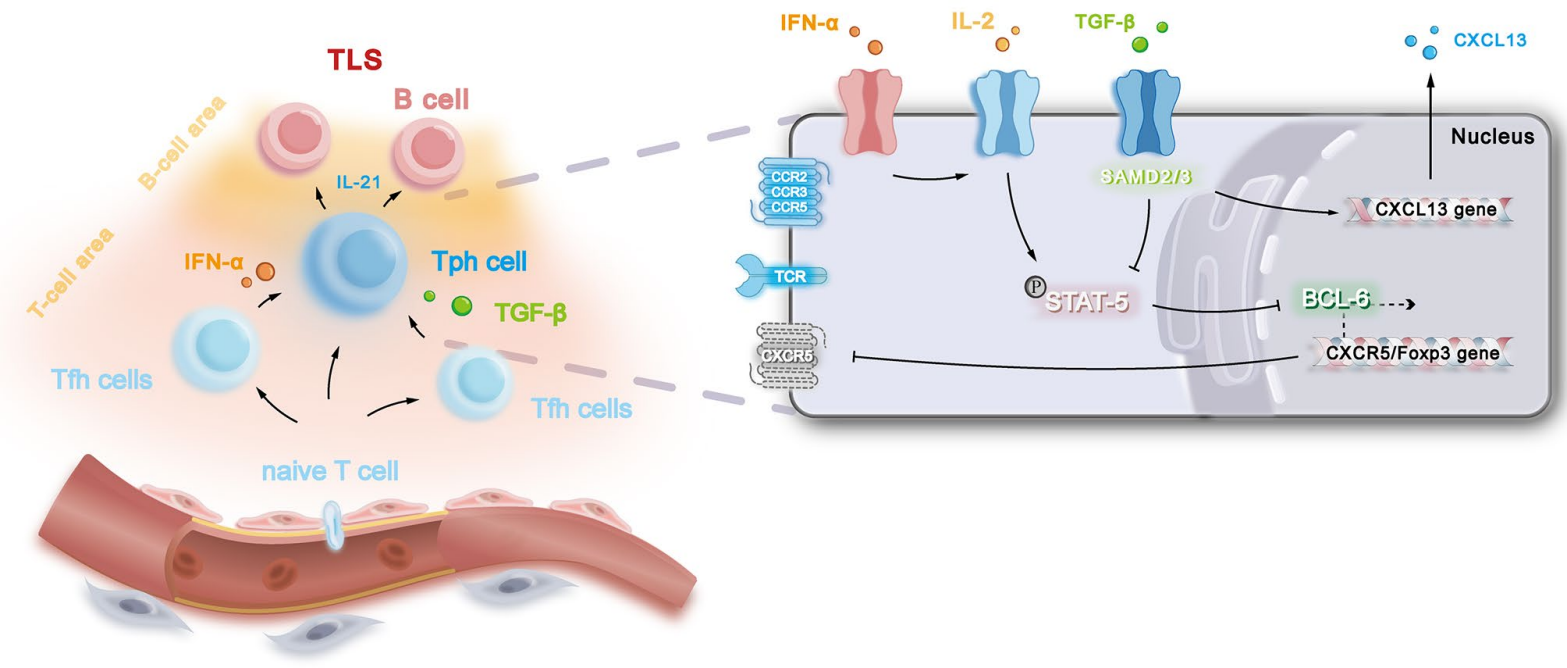
Tph cells typically migrate to and execute crucial functions within tertiary lymphoid structures (TLS). At these sites, Tph cells may differentiate from various precursor cells, including naïve T cells, Th2 cells, Th17 cells, and Tfh cells. Within TLS, IFN-α markedly enhances T-cell responsiveness to IL-2/STAT5 signaling, resulting in the downregulation of CXCR5 expression. Concurrently, TGF-β plays an indispensable role in promoting CXCL13 expression in Tph cells

Regarding Tfh cell phenotype, high PD-1, CXCR5, and ICOS expression distinguish them from Tph cells (Yoshitomi and Ueno 2021). Additionally, Tfh cells possess CD40 ligand (CD40L), tumor necrosis factor receptor superfamily member 4 (OX40), and T cell immunoreceptor with immunoglobulin and ITIM domain (TIGIT) (Wei and Niu 2023). (Fig. 1)

## The general function of Tph/Tfh cells in autoimmune diseases

The lack of CXCR5 potentially hampers their migration towards CXCL13-enriched regions (Pan et al. 2022). However, the expression of chemokine receptors such as CCR2 and CCR5 promotes their migration toward peripheral inflammatory tissues rich in diverse chemokines, resulting in a preferential localization within tertiary lymphoid structures (TLS) (Sato et al. 2023). After reaching TLS, Tph cells also wield the capability to elicit heightened expression of T-bet within B cells, thereby fostering the differentiation of CD21low B cell subtypes. Furthermore, Tph cells exhibit the capacity to extend T-cell assistance using costimulatory interactions involving CD40L and IL-21 receptors (Keller et al. 2021). Within these specialized microenvironments, Tph cells engage in intricate crosstalk with B cells and other T cells, thereby triggering the release of immune modulators and intensifying the inflammatory cascade. In summary, Tph cells play a critical role in fostering the activation of autoantibody-secreting B cells, secretion of cytokines leading to tissue damage, and recruitment of diverse T cell subsets (Huang et al. 2023; Christophersen et al. 2019; Marks and Rao 2022; Yoshitomi 2022).

As for the function of Tfh cells, antigens presented by DCs and costimulation via ICOS-L and IL-6 from DCs induce Bcl6 expression, committing the T cells to the Tfh lineage in germinal centres (Jogdand et al. 2016). Within germinal centres, Tfh cells directly interact with B cells, providing costimulation via the CD40-CD40L interaction and producing the cytokine IL-21 to drive B cell proliferation (Crotty 2011).

## Tph and IgA nephropathy

IgA nephropathy (IgAN) is a significant renal disorder that commonly affects the younger demographic and has the potential to progress into chronic kidney disease, including end-stage renal failure (Rajasekaran et al. 2021). It is characterized by the renal accumulation of immunoglobulin A and subsequent inflammatory responses, engendering renal tissue damage (Du et al. 2023). The pathophysiology of IgAN is multifaceted and involves the immune system, characterized by the accumulation of IgA-containing immune complexes in the glomerular mesangium (Knoppova et al. 2021). Although the precise etiology of IgAN remains elusive, current literature suggests that the four-strike doctrine is the predominant hypothesis (IgA nephropathy 2016). In addition, Tph cells have been identified as critical contributors to the pathogenesis of IgAN.

The initial stage of the four-strike doctrine involves the over-activation of IgA1-producing cells, resulting in the production of galactose-deficient IgA (Gd-IgA) antibodies in afflicted individuals (Gentile et al. 2023). Subsequently, Tph cells, elevated in patients with IgAN, can infiltrate the glomerular mesangium. TGF-β overexpression in renal tubular endothelial cells and augmented IFN levels in the blood can stimulate the differentiation of naive T cells into Tph cells (Goumenos et al. 2002; Shan et al. 2022; Zheng et al. 2017; Tanemura et al. 2022a, b). It is conceivable that pDCs induced by Toll-like receptor 9 (TLR9) activation play a role in the heightened activation of their secreted IFN-α, known as the pDC-IFN-α axis, in individuals with IgAN. Additionally, this mechanism may further stimulate the generation of Tph cells in the affected microenvironment (Zheng et al. 2017). In the glomerular mesangium, Tph cells demonstrate increased RNA levels of IL-21/CXCL13 and upregulation of surface proteins, including PD-1, CCR2, CCR3, and CXCR1 (Wang et al. 2020). Once situated in the mesangium, Tph cells interact with CD38^+^ B cells through CD84/CD40, instigating the production of secreted anti-Gd-IgA antibodies through the secretion of IL-21 and CXCL13 (Tangye 2017). These antibodies bind to CD138^+^ B cells, and heightened levels of IgG4 serve as a key indicator of autoimmune hepatitis, leading to the degradation and exacerbation of IgAN (Zhang et al. 2014). In the subsequent phase, the anti-Gd-IgA antibodies attach to Gd-IgA, generating large molecular weight immune complexes that deposit in the mesangium. Ultimately, the final stage involves the immune complexes inducing the proliferation of mesangial cells and massive production of the matrix, ultimately contributing to the exacerbation of IgAN (Chang and Li 2020). Furthermore, IgAN patients exhibiting tertiary lymphoid structures (TLS) showed a notable elevation in the counts of Tfh and Tph cells. Tph cells localized within the TLS may concurrently release IL-21 alongside Tfh cells, thereby fostering the development of renal fibrosis (Luo et al. 2023). CD4^+^ T cells infiltrating renal tissues may play a pivotal role in renal injury by activating B cells in situ, thereby contributing significantly to the pathogenesis of IgAN (Du et al. 2022). In summary, the four-strike doctrine is widely accepted as the pathogenic mechanism of IgAN, with Tph cells playing a crucial role in the pathogenesis and disease progression.

## Tph and IgG4-related renal disease

IgG4-related disease (IgG4-RD) entails elevated levels of IgG4 antibodies, instigating kidney tissue impairment (Sánchez-Oro et al. 2019). The etiology of IgG4-related kidney disease is intricately linked with both genetic and immune factors involving T cells (Mbengue et al. 2021). This disorder is marked by substantial T-cell infiltration in the renal tubular interstitium along with the presence of IgG4-producing B cells, leading to the formation of antigen-antibody complexes and subsequent fibrotic changes (Saeki and Kawano 2014). However, the precise mechanism by which IgG4 secretion by B cells influences the progression of renal fibrosis remains an area of active investigation (Kawano et al. 2023).

In IgG4-related disease patients, the quantity of Tph cells within the peripheral blood of IgG4-related disease patients markedly surpasses that of healthy counterparts (Kamekura et al. 2018; Mancuso et al. 2021). These cells are typified by elevated levels of CD25, CD38, and TIGIT, along with diminished levels of T-bet, but Tfh-like cells expressed more CCR7 and CD127 than Tph cells (Zhang et al. 2022). Notably, CCR7 is known to play a pivotal role in cell migration to lymphoid organs (Hong et al. 2022), while CD127 is a cytokine receptor for IL-7, a critical factor for T cell survival and steady-state proliferation (Chen et al. 2021). Consequently, Tph-like cells may have a reduced ability to migrate to secondary lymphoid organs when compared to Tfh cells. However, the heightened expression of CX3CR1 chemokine receptors on Tph cells facilitates their migration to inflammatory tissues (Yabe et al. 2021). Thus, a theoretical basis suggests that Tph cells may actively facilitate the onset and perpetuation of IgG4-RD through the secretion of chemokines and engagement of costimulatory molecules. These molecular mediators recruit Tfh cells and B cells to the inflamed sites, potentially amplifying the pathological processes inherent to IgG4-RD (Cargill et al. 2020). Additionally, the observed positive correlation between Tph cells and the frequency of plasma cells present in secondary lymphoid organs suggests a potential role for Tph cells in promoting the differentiation of naive B cells (Zhang et al. 2022). Tph cells establish interactions with B cells through TIGIT, leading to an upregulation of IL-21 expression and subsequent stimulation of B cell differentiation into plasma cells via IL-21 signaling. Silencing of TIGIT in Tph cells results in a reduction of IL-21 expression and impedes the differentiation of B cells into plasma cells. Tph cells, particularly TIGIT^+^ Tph cells, exhibit potential as discernible markers for assessing the activity of IgG4-related disease (Ji et al. 2023; Akiyama et al. 2021). The proportion of Tph cells positively correlates with serum IgG4 levels, the extent of organ involvement, and the percentage of CD11c^+^CD21^−^ B cells (Kamekura et al. 2022). Moreover, Tph cells produce CXCL13, which attracts CXCR5^+^ Tfh cells, and together with Tfh cells, form ectopic lymph node-like structures that contribute to the initiation of inflammation and the perpetuation of chronic fibrositis. Furthermore, Tph cells expressing CX3CR1 demonstrate cytotoxicity, leading to the apoptosis of vascular endothelial and ductal epithelial cells by releasing granzyme and perforin. Significantly, these cells exhibit heightened expression of CX3CL1, a ligand for CX3CR1, within the affected organs of patients with IgG4-RD (Yabe et al. 2021; Kamekura et al. 2019). Tph cells, in conjunction with Th2 cells, Treg cells, and Tfh cells, actively foster the differentiation of naïve B cells into plasma cells. This process ultimately intensifies the manifestations of IgG4-RD (Liu et al. 2020).

**Fig. 2:**
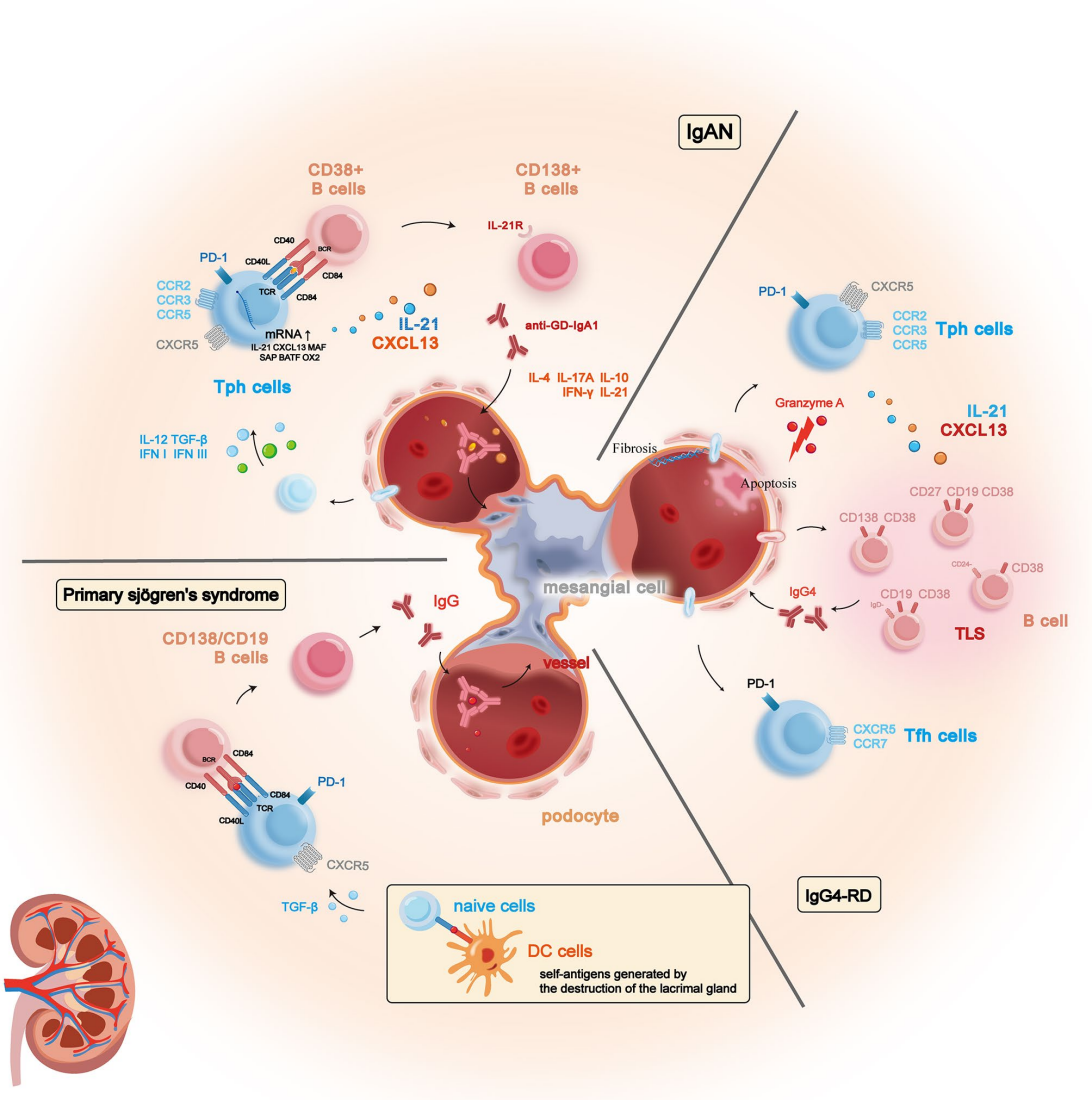
The involvement of Tph cells in the pathogenesis of IgA nephropathy, pSS, and IgG4 nephropathy. In these conditions, Tph cells are robustly activated and expanded within peripheral inflammatory tissues, where they secrete cytokines such as IL-21 and CXCL13 to stimulate the activation of numerous autoantibody-producing B cells. This results in the deposition of large quantities of antigen-antibody complexes, leading to an increase in thylakoid cell and stromal cell numbers. Furthermore, in IgG4 nephropathy, Tph cells can also exert cytotoxic effects, causing necrosis of glomerular epithelial cells and promoting mesangial cell proliferation and fibrosis

In conclusion, Tph cells have been demonstrated to exert a crucial role in the pathogenesis of IgG4-related nephropathy through their ability to activate B cells and recruit Tfh cells, ultimately contributing to disease exacerbation. (Fig. 2)

## Tph and diabetic nephropathy

DN represents a microvascular complication of diabetes mellitus that manifests as a progressive decline in renal function (Kanwar et al. 2011). The pathogenesis of DN is intricately associated with the presence of hyperglycemia. Hyperglycemia represents a primary etiological factor in the pathogenesis of DN. Hyperglycemia exerts its deleterious effects by compromising the integrity of the renal microvasculature, disrupting normal kidney function (Papadopoulou-Marketou et al. 2017). Furthermore, hyperglycemia also impacts the glomerular filtration membrane, rendering it more permeable and resulting in the excretion of human molecules, including proteins, into the urine. Continued renal exposure to these substances may lead to kidney damage and ultimately contribute to the development and progression of DN (Alsharidah 2022). Hence, investigating the specific mechanisms underlying the involvement of Tph cells in hyperglycemia is of paramount importance in the context of elucidating the pathogenesis of DN. In children newly diagnosed with type 1 diabetes (T1D) who display multiple autoantibodies, a higher frequency of circulating Tph cells with elevated TIGHT expression was observed (Ekman et al. 2019). TIGHT signaling in these cells may promote a tolerogenic phenotype in DCs (Annese et al. 2022), which may contribute to the exacerbation of autoimmune disease development (Kim et al. 2021). Furthermore, there is an indication that IL-21 is not solely generated by CXCR5^+^ T follicular helper cells within the pancreatic islets of individuals with T1D since Tph cells may also contribute to its production (Viisanen et al. 2016).

Moreover, Tph overexpression in animal models is associated with a reduction in the frequency of spleen naive B cells and an increase in non-conventional memory CD27^−^IgD^−^ B cells (Vecchione et al. 2022). Under certain stimuli, CD27^−^IgD^−^ B cells can differentiate into antibody-secreting cells (ASCs) that produce immune complexes capable of infiltrating the glomerulus (Beckers et al. 2023; Vandamme and Kinnunen 2020), exacerbating thylakoid expansion and glomerular basement membrane thickening, thus contributing to the progression of DN (Smith et al. 2017). Moreover, the involvement of Tfh cells in DN has been documented, evidenced by an elevated count of CD4^+^CXCR5^+^PD-1^+^ Tfh cells in DN patients. This increase exhibits a negative correlation with estimated glomerular filtration rate (eGFR) and a positive correlation with 24-hour urine protein concentration. However, the precise mechanism of Tph cells in this context remains under exploration (Zhang et al. 2016).

Proposedly, after the induction of islet antigen-specific Tfh cells in pancreatic lymph nodes, certain Tfh cells undergo a transition marked by the loss of CXCR5 expression and an upregulation of chemokine receptors such as CCR2, CCR5, and CX3CR1, transforming into Tph cells that exit lymph nodes. These Tph cells are attracted to inflamed pancreatic islets by chemokines CCL2, CCL5, and CX3CL1. Furthermore, Tph cells produce CXCL13, attracting B cells to inflamed pancreatic islets and generating IL-21, fostering the maturation of B cells. This process culminates in the localized production of autoantibodies (AAbs). Additionally, IL-21, generated by Tph cells, may support the proliferation and survival of cytotoxic CD8 T cells, the predominant subset of immune cells infiltrating inflamed pancreatic islets (Vandamme and Kinnunen 2020).

In summary, highly amplified Tph cells have been implicated in the pathogenesis of DN through multiple mechanisms. Specifically, Tph cells may directly impair islet function by promoting DC immune tolerance and CD8^+^ T cell activation, leading to hyperglycemia in affected kidneys. Alternatively, Tph cells may contribute to the production of double-negative B cells and antibody-secreting cells, resulting in the secretion of autoantibodies and the formation of larger immune complexes that exacerbate glomerular injury.

## Tph and ANCA vasculitis-affected kidneys

ANCA-associated vasculitis (AAV), characterized by vascular inflammation and necrosis, frequently leads to glomerulonephritis and consequential renal dysfunction (Austin et al. 2022). AAV is a chronic autoimmune disease characterized by recurrent episodes of small vessel inflammation, affecting primarily the respiratory tract and kidneys (Sunderkötter et al. 2018). AAV is a major cause of rapidly progressive glomerulonephritis (GN), often presenting as pauci-immune necrotizing crescentic GN and associated with high morbidity, including a high risk of progression to end-stage kidney disease (ESKD) and increased mortality (Scurt et al. 2022). The pathogenesis of AAV is currently believed to involve the activation of neutrophils by antibodies to ANCA. These antibodies bind to ANCA antigens located at the basement membrane of glomerular vessels, leading to glomerular vascular damage via neutrophil respiratory bursts and other mechanisms (Jennette and Nachman 2017).

Elevated circulating Tph cell counts have been observed in patients with active AAV, and these cells have been found to exacerbate the development and progression of ANCA-associated vasculitis through the production of cytokines such as TNF-α, IL-4, IL-21, and IL-12, as well as the presence of elevated serum MPO-ANCAs concentrations (Krajewska Wojciechowska et al. 2021; Liu et al. 2022). Conversely, serum IL-10 has been found to exhibit a negative correlation with circulating Tph cells in active AAV patients. A reduction in IL-10 is anticipated to contribute to the escalation of the disease progression (Liu et al. 2022; Diefenhardt et al. 2018). Furthermore, a study showed that the percentage of memory T cells from MPO-ANCA-associated vasculitis patients was significantly higher than that of normal controls (Hirayama et al. 2010). However, in a recent study, no significant difference was observed in the frequency of Tph cells between patients diagnosed with granulomatosis with polyangiitis, microscopic polyangiitis, and healthy controls (London et al. 2021). This finding contrasts previous studies, which demonstrated an increased frequency of Tph cells in patients with active AAV and suggested their contribution to the pathogenesis of the disease. The differences in the disease status and treatment regimens between the patient cohorts in the different studies may have contributed to the discrepant results. Further investigation is needed to elucidate the role of Tph cells in the pathogenesis of polyangiitis and microscopic polyangiitis.

## Tph and primary sjögren’s syndrome

Primary Sjögren’s syndrome (pSS), an autoimmune disorder affecting exocrine glands, including the lacrimal glands, can also involve the kidneys, resulting in reduced renal function due to autoantibody-mediated damage (Goules et al. 2019). The pSS is a multifaceted autoimmune disease associated with systemic complications and lymphoma development (Manfrè et al. 2022). It is characterized by T cell-mediated hyperactivation of B cells and can affect multiple organs, including the kidneys (Aiyegbusi et al. 2021). Renal involvement is a common feature of pSS (Luo et al. 2019).

Following the destruction of the lacrimal gland, autoantigens are generated and presented by DCs to naive T cells, which can lead to their differentiation into Tph cells. This process is significant as Tph cells have been implicated in the pathogenesis of various autoimmune disorders, including Sjögren’s syndrome (Verstappen et al. 2021). Enriching Tph cells in the peripheral blood and labial gland of patients with pSS suggests their involvement in developing this disease. Tph cells were significantly associated with disease activity scores, including ESSDAI scores, IgG, ESR, IL-21, and anti-SSA antibody levels (Chen et al. 2022). Key pathogenic players in the immunopathology of pSS include IL-21 and the ICOS costimulatory pathway and IL-21/IFN-γ double-production (Pontarini et al. 2020a, b); thus, IFN-γ may also play a role in the disease (Sato et al. 2022). In addition, TGF-β is believed to contribute to the differentiation of Tph cells, which have been shown to promote the activation of B cells, particularly CD138/CD19 plasma cells, in patients with pSS (Shan et al. 2022; Dupré et al. 2021; Maślińska et al. 2019). In addition to generating antigen-antibody complexes directed against the lacrimal gland’s autoantigens, activated Tph cells, B cells, and plasma cells can also infiltrate the renal interstitium, resulting in the onset of tubulointerstitial nephritis (François and Mariette 2016).

## Tph and lupus nephritis

Lupus nephritis (LN), an aggressive manifestation of systemic lupus erythematosus (SLE), manifests as severe renal inflammation and damage (Bhargava et al. 2023). SLE is a complex autoimmune disease characterized by its chronic and recurrent nature, encompassing a diverse array of symptoms ranging from mild to severe, with life-threatening manifestations. The pathogenesis of SLE is intricately associated with the presence of autoantibodies, immune complexes, and aberrant immune responses (Basta et al. 2020). Despite extensive research efforts, the precise mechanisms underlying the development and progression of SLE remain elusive and challenging to ascertain definitively (Crow 2023). Among the various organ manifestations observed in SLE, lupus nephropathy represents a particularly grave form of glomerulonephritis, posing significant clinical implications and requiring comprehensive management strategies (Anders et al. 2020).

In previous investigations, the existence of Tph cells in human lupus nephritis samples has been confirmed through meticulous analysis utilizing single-cell RNA sequencing (Arazi et al. 2019). Notably, an augmented population of Tph cells with a distinctive PD-1^hi^CXCR5^−^ICOS^+^CD38^+^HLA-DR^+^MHC-II^+^ phenotype has been discerned in the peripheral blood of patients diagnosed with SLE (Choi et al. 2015; Lin et al. 2019). Based on the expression profiles of CXCR3 and CCR6, Tph cells can be categorized into three distinct subtypes: CXCR3^+^CCR6^−^Tph (referred to as Tph1), CXCR3^−^CCR6^−^Tph (referred to as Tph2), and CXCR3^−^CCR6^+^Tph (referred to as Tph17) (Makiyama et al. 2019a, b). However, in combination, the expression of CXCR5 and ICOS was found to be minimally detectable among CCR6^+^IL-7R^+^ T cells. This observation suggests a limited representation of CXCR5 and ICOS co-expressing cells within the CCR6^+^IL-7R^+^ T cell population (Facciotti et al. 2020).

Tph cells can induce the differentiation of naive B cells into CD27/CD38 double-positive plasma cells in a manner that relies on the presence of IL-21 and the transcription factor MAF (Lin et al. 2019; Bocharnikov et al. 2019; Deng et al. 2015). This process exhibits a positive correlation with the level of disease activity. Furthermore, the production of IL-21 by Tph cells directly exacerbates the pathological processes associated with lupus (Herber et al. 2007). Additionally, the elevated expression of granzyme B, a protease enzyme, in Tph cells plays a pivotal role in the deterioration of renal function in lupus nephritis (Bocharnikov et al. 2019; Kok et al. 2017).

Furthermore, in individuals diagnosed with SLE, the release of mitochondrial DNA (mtDNA) from neutrophils triggers the activation of pDCs, which may induce the differentiation of naive T cells into Tph cells. Subsequently, the activated Tph cells play a role in the reactivation of naive B cells by employing interleukin-10 (IL-10) and succinate, leading to the differentiation of CD18^+^CD21^+^CD11c^−^ plasma cells and the secretion of IgG antibodies (Caielli et al. 2019; Biswas et al. 2022). Notably, recent investigations have identified a distinct subset of plasma cells characterized by the CD18^+^CD21^−^CD11c^+^ phenotype, known as ABC cells, which are closely associated with the pathogenesis of LN (Sachinidis et al. 2020).

**Fig. 3:**
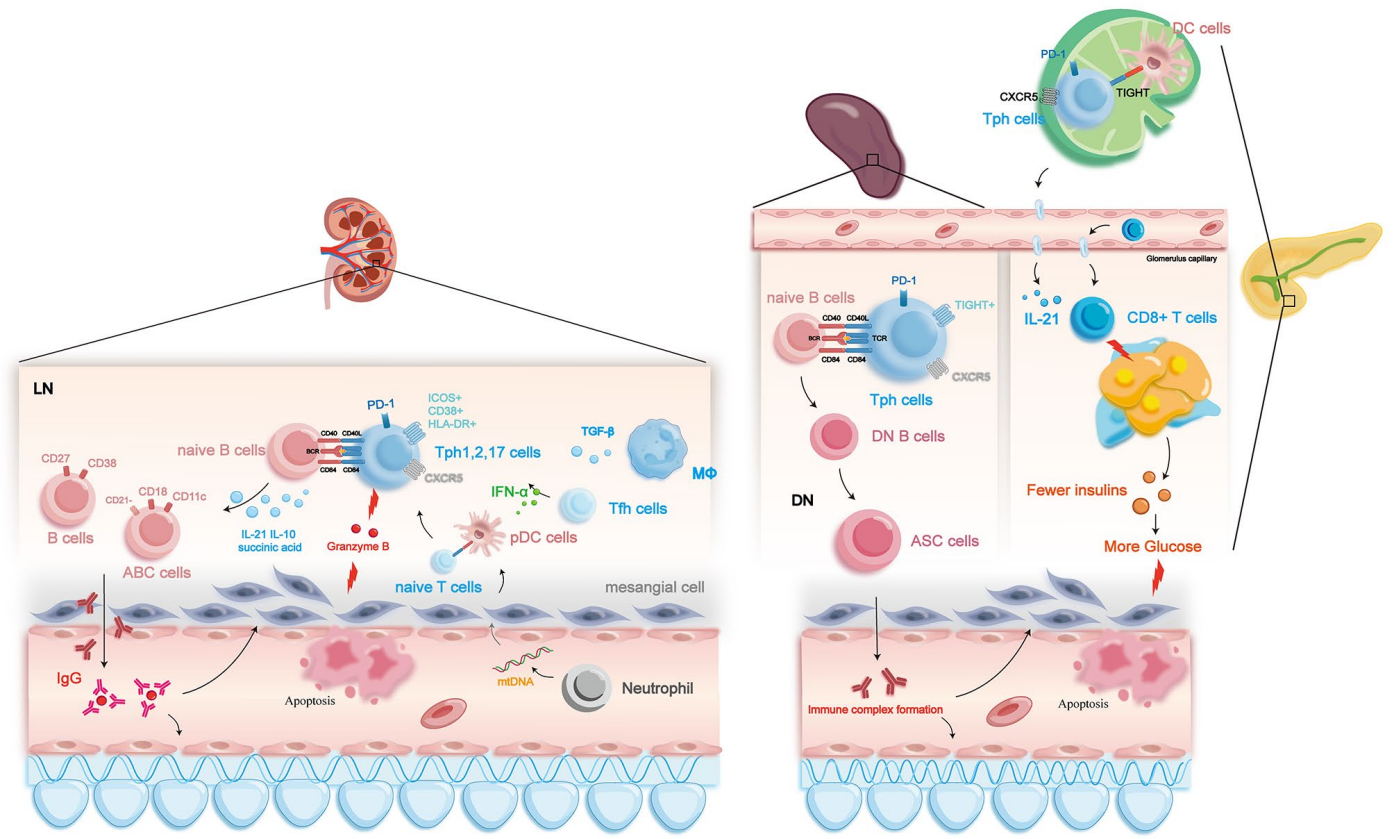
The role of Tph cells in the pathogenesis of lupus nephritis and diabetic nephropathy. In lupus nephritis, pDC cells and macrophages can provide the necessary cytokines to facilitate the differentiation of T cells into Tph cells. These Tph cells can then secrete IL-21, succinic acid, and other factors to promote the differentiation of autoantibody-producing B cells. Additionally, Tph cells in this context exhibit cytotoxic activity. In diabetic nephropathy, an autoimmune response at the pancreatic islets leads to the proliferation of Tph cells within the islets, resulting in their destruction. This impairs insulin secretion, leading to hyperglycemia and subsequent renal involvement

Elevated levels of IFN-α have been observed in patients diagnosed with SLE, a complex autoimmune disorder (Niewold 2011). The upregulation of IFN-α has been implicated in differentiating Tfh cells into Tph cells. Within this context, Tph cells in the presence of elevated IFN-α demonstrate a heightened sensitivity to IL-2 stimulation. Consequently, activating the IL-2 signaling pathway results in the preferential binding of STAT5 to the BCL6 locus at the expense of STAT1. This preferential binding pattern negatively impacts the concurrent expression of histone H3 lysine 4 trimethylation and subsequently leads to the downregulation of CXCR5 expression, an essential marker for Tph cells (Jiang et al. 2022). Furthermore, the elevated levels of IFN-λ in SLE patients (Barnas et al. 2021) contribute to the differentiation of Tph cells, along with B cells, thereby exacerbating the pathological progression of the disease (Tanemura et al. 2022a, b). However, it is noteworthy to mention that one study reported no significant alterations in Tph cell levels following rituximab treatment, indicating a potential lack of responsiveness of Tph cells to disease activity modulation (Faustini et al. 2022). (Fig. 3)

## Tph and cGVHD affected the kidney

Chronic graft-versus-host disease (cGVHD) stands as a significant and formidable complication following transplantation, with its primary impact directed towards the skin, gastrointestinal tract, liver, lungs, and mucosal surfaces (Li and Zhang 2019). The pathogenesis of cGVHD entails intricate interactions involving immune cells from both the donor and the host (Nasr et al. 2022). It has come to light that GVHD could potentially contribute to various glomerular pathologies. Moreover, cGVHD has the capacity to incite renal inflammation reminiscent of SLE, as evidenced by a murine model (Zhang et al. 2023; He et al. 2012). Consequently, a thorough exploration into the role of Tph cells within the context of cGVHD is paramount in our quest to alleviate renal ailments triggered by cGVHD.

Among individuals grappling with cGVHD, a discernible expansion of Tph cells has been observed, accompanied by the heightened expression of surface markers such as PD-1, TIGHT, ICOS, and HLA-DR. However, markers like CD160, LAG3, or 2B4 have not exhibited an upregulation. Furthermore, an examination of the intracellular domain of Tph cells has revealed marked elevations in the expression of transcription factors, including BLIMP1, MAF, and IRF4. This subgroup of Tph cells also displays a substantial secretion of IFN-γ, IL-21, and IL-4 while not displaying an upregulation of CXCL13 and IL-2. An additional noteworthy phenomenon is the capacity of Tph cells to migrate from the circulatory system to anatomical locales such as the liver or lungs, where they undergo a transition into T regulatory helper (Trh) cells. Within the inducible bronchus-associated lymphoid tissue (iBALT), CD4^+^ cells predominantly present as PSGL1^lo^CD69^+^ Trh cells, engaging in intricate interactions with B cells. Notably, the process hinges on the IL-21R-BCL6 signalling pathway, in tandem with the pivotal role of T-bet, orchestrating the differentiation and expansion of both Tph and Trh cells. The influence of IL-21 extends to developing T cells, prompting their differentiation into Tph and Trh cells, subsequently intensifying the development and proliferation of high-affinity centre B cells marked by Fas^+^GL7^+^IgG^+^IgD^−^ memory B cells and CD27^+^CD138^+^ plasma cells. This cascading effect leads to an elevation in the overall concentration of total IgG alongside anti-double-stranded DNA (dsDNA) IgG, thereby exacerbating the autoimmune inflammatory response (Kong et al. 2022; Chen et al. 2023).

## Tph and acute virus-affected kidney

The influence of the COVID-19 pandemic on renal health has emerged as a prominent subject of investigation. Beyond its respiratory impact, the immune response incited by the virus possesses the potential to inflict enduring and severe damage upon various organs, including the kidneys (Dadson et al. 2020). Moreover, viral infections have been implicated in developing kidney-related disorders (Iwata and Tanaka 2022). Thus, comprehending the intricate role played by Tph cells in acute viral infections carries substantial scientific significance. In COVID-19 patients, the phenotype of Tph cells is typified by the distinctive markers PD1^hi^CXCR5^−^CD38^+^HLA-DR^+^ and CTLA4^lo^. This Tph cell population displays a notable male-biased cellular network configuration (Søndergaard et al. 2023). Within the acute phase of COVID-19, a marked surge in the Tph cell count has been observed, displaying a direct correlation with the frequency of plasma cells. Noteworthy is the array of genes associated with Tfh cell’s function expressed by Tph cells, encompassing MAF, TIGIT, SLAMF6, and IL-21. Phenotypically, these Tph cells exhibit heightened levels of CCR5 and CCR2 compared to conventional Tfh. These Tph cells can secrete both IL-21 and CXCL13, factors pivotal in steering B cell differentiation toward the plasma cell lineage (Asashima et al. 2023).

Significantly, the secretion of IFN-γ by Tph cells, at appropriate levels, hinges on the expression of tissue-homing receptors such as CXCR3 on plasma cells. This phenomenon further reinforces the directed congregation of plasma cells within tissues. In line with this observation, a substantial positive correlation between CXCR3 + plasma cells and Tph cells is evident. These activated plasma cells can synthesize immunoglobulins, most notably IgG (Asashima et al. 2021).

## Tph and autoimmune liver disease affected the kidney

Primary Biliary Cirrhosis (PBC) stands as a chronic inflammatory autoimmune disorder, the intricacies of its onset being orchestrated by a multifaceted interplay between genetic predispositions and environmental factors (Xie et al. 2016). Within the domain of PBC, an observable renal manifestation takes the form of asymptomatic Distal Renal Tubular Acidosis (DTA), bearing the potential ramifications of inciting tubulointerstitial nephritis (Bansal et al. 2012; Komatsuda et al. 2010).

Notably, in PBC patients, a notable augmentation in the population of Tph cells marked by heightened expressions of CD28 and TIGHT is evident within the peripheral blood milieu. Following treatment initiation, a discernible trend towards gradually reducing these Tph cells comes into view. Of particular import is the intimate association between these two distinct subsets of cells and the activation status of CD19^+^CD38^hi^CD138^+^ plasma cells. Additionally, this association extends to the intricate orchestration of the transformation process, wherein CD27^−^IgD^+^ primary B cells evolve into the CD27^+^IgD^−^ profile characteristic of memory B cells, concomitantly contributing to an elevated synthesis of IgG antibodies. A compelling observation is the significant positive correlation between ICOS^+^ Tph cells and both IgG antibody levels and the serum marker AMA-M2 within the context of PBC patients (Yong et al. 2021).

Autoimmune Hepatitis (AIH) emerges as a persistent inflammatory disorder characterized by elevated levels of gamma globulins, the presence of autoantibodies in serum, and histopathological indications of interface hepatitis. Untreated, this disorder possesses the potential to escalate into cirrhosis and advanced liver dysfunction. Moreover, compelling research underscores a robust linkage between autoimmune hepatitis and renal afflictions (Muratori et al. 2023).

In individuals who have autoimmune hepatitis, Tph cells emerge as the principal peripheral reservoir housing autoreactive CD4 T cells that specifically target Soluble Liver Antigens (SLA). Notably, the co-expression of PD-1 and CD38 within CD45RA^−^CXCR5^−^CD127^−^CD27^+^ T cell subsets assume a pivotal role as an immunologically informative marker of active autoimmune hepatitis. These Tph cells hold the capacity to guide B cells through pathways reliant on IL-21, thereby steering their differentiation trajectory towards the plasma cell lineage. Remarkably, the population of these Tph cells demonstrates a positive correlation with the levels of IgG in autoimmune hepatitis patients (Renand et al. 2020).

## Conclusion

The role of Tph cells is extremely important in renal diseases. A large body of evidence suggests that in the development of autoimmune diseases, the activation of Th0 cells or the downregulation of Tfh cells’ CXCR5 expression in peripheral blood cells leads to the generation of Tph cells. Tph cells influence the differentiation of other Th cells and B cells by producing CXCL13 and various leukocyte cytokines in different regions, thereby playing an indispensable role in the pathogenic mechanisms of various autoimmune renal diseases and other nephritis. Inhibiting the excessive activation of Tph cells to reduce the release of pro-inflammatory factors and suppress excessive immune activity of other T cells is a potential approach to alleviate autoimmune diseases.

## Data Availability

Not applicable.

## Abbreviations

AAV: ANCA-associated vasculitis
AIH: Autoimmune Hepatitis
ASCs: Antibody-secreting cells
CCR2: C-C chemokine receptor 2
CCR3: C-C chemokine receptor 3
CCR5: C-C chemokine receptor 5
CCR7: C-C chemokine receptor 7
cGVHD: Chronic graft-versus-host disease
CX3CR1: C-X3-C motif chemokine receptor 1
CXCR5: C-X-C motif chemokine receptor 5
DCs: Dendritic cells
DN: Diabetic nephropathy
ESKD: End-stage kidney disease
Gd-IgA: Galactose-deficient IgA
GN: Glomerulonephritis
IFN-α: Interferon-alpha
IgAN: IgA nephropathy
IgG4-RD: IgG4-related disease
IL-21: Interleukin-21
IL-2: Interleukin-2
LN: Lupus nephritis
PBC: Primary Biliary Cirrhosis
PD-1: Programmed cell death protein 1
pSS: Primary Sjögren’s syndrome
SLE: Systemic lupus erythematosus
STAT5: Transcription 5
T1D: Type 1 diabetes
Tfh cells: T follicular helper T cells
TGF-β: Transforming growth factor beta
TLS: Tertiary lymphoid structures
Tph cells: T peripheral helper cells
Trh cells: T regulatory helper cells
