# Correction: Occurrence and role of Tph cells in various renal diseases

**DOI:** 10.1186/s10020-024-00974-w

**Published:** 2024-11-13

**Authors:** Junyi Ren, Kuai Ma, Xiangheng Lu, Haoyu Peng, Jia Wang, Moussa Ide Nasser, Chi Liu

**Affiliations:** 1 School of Medicine, University of Electronic Science and Technology of China, Chengdu, China; 2 Department of Nephrology, Osaka University Graduate School of Medicine, Osaka, Japan; 3 Department of Ophthalmology, West China Hospital, Sichuan University, Chengdu, Sichuan China; 4 General Practice Center, Sichuan Provincial People’s Hospital, Sichuan Academy of Sciences, University of Electronic Science and Technology, Chengdu, 610072 China; 5 Department of Cardiac Surgery, Guangdong Cardiovascular Institute, Guangdong Provincial People’s Hospital (Guangdong Academy of Medical Sciences, Southern Medical University, Guangzhou, 510100 Guangdong China; 6 Department of Nephrology, Institute of Nephrology, Sichuan Provincial People’s Hospital, Sichuan Clinical Research Centre for Kidney Diseases, Chengdu, China

**Correction: Molecular Medicine (2024) 30:174**

10.1186/s10020-024-00919-3

Following publication of the original article [1], the authors reported that Figs. 1 and 3 should be swapped, and Fig. 2 should be replaced.

The incorrect Fig. 2 is:

**Fig. 2:**
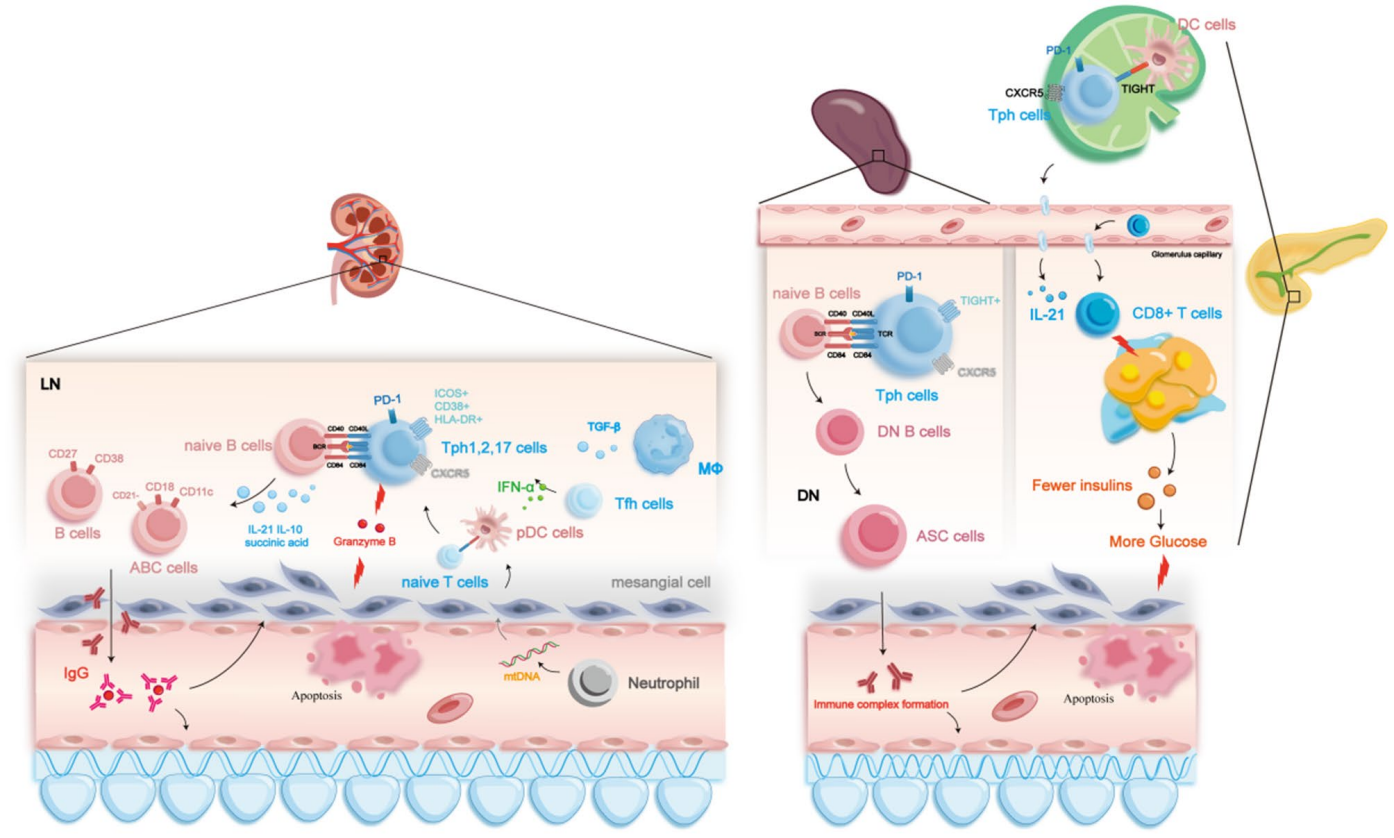
The involvement of Tph cells in the pathogenesis of IgA nephropathy, pSS, and IgG4 nephropathy. In these conditions, Tph cells are robustly activated and expanded within peripheral inflammatory tissues, where they secrete cytokines such as IL-21 and CXCL13 to stimulate the activation of numerous autoantibody-producing B cells. This results in the deposition of large quantities of antigen-antibody complexes. Furthermore, in IgG4 nephropathy, Tph cells can also exert cytotoxic effects, causing necrosis of glomerular epithelial cells and promoting mesangial cell proliferation and fibrosis

The correct Fig. 2 is:

**Fig. 2:**
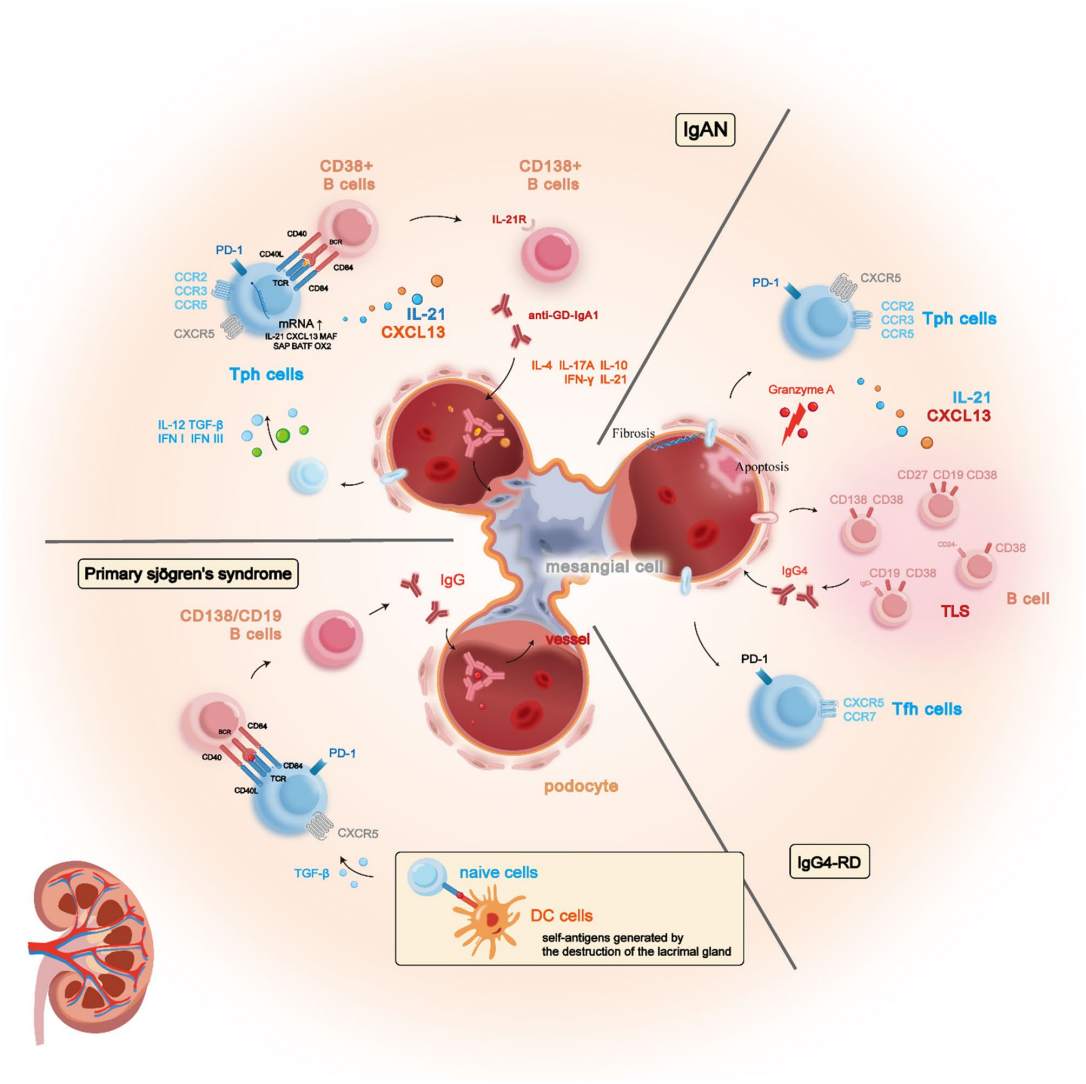
The involvement of Tph cells in the pathogenesis of IgA nephropathy, pSS, and IgG4 nephropathy. In these conditions, Tph cells are robustly activated and expanded within peripheral inflammatory tissues, where they secrete cytokines such as IL-21 and CXCL13 to stimulate the activation of numerous autoantibody-producing B cells. This results in the deposition of large quantities of antigen-antibody complexes. Furthermore, in IgG4 nephropathy, Tph cells can also exert cytotoxic effects, causing necrosis of glomerular epithelial cells and promoting mesangial cell proliferation and fibrosis

The original article has been corrected.
